# Implications of PI3K/AKT/PTEN Signaling on Superoxide Dismutases Expression and in the Pathogenesis of Alzheimer’s Disease

**DOI:** 10.3390/diseases6020028

**Published:** 2018-04-20

**Authors:** Satoru Matsuda, Yukie Nakagawa, Ai Tsuji, Yasuko Kitagishi, Atsuko Nakanishi, Toshiyuki Murai

**Affiliations:** 1 Department of Food Science and Nutrition, Nara Women’s University, Kita-Uoya Nishimachi, Nara 630-8506, Japan; yukiie0028@yahoo.co.jp (Y.N.); 1452rtob@gmail.com (A.T.); y_kitagishi@live.jp (Y.K.); 2 Department of Food and Nutrition, Faculty of Contemporary Human Life Science, Tezukayama University, Nara 631-8501, Japan; gah00635@nifty.com; 3 Department of Microbiology and Immunology, Graduate School of Medicine, Osaka University, Suita 565-0871, Japan; pi3kp10@outlook.jp

**Keywords:** Alzheimer’s disease, PI3K, AKT, PTEN, n-3 PUFAs, PPAR, superoxide dismutase, reactive oxygen species

## Abstract

Alzheimer’s disease is a neurodegenerative sickness, where the speed of personal disease progression differs prominently due to genetic and environmental factors such as life style. Alzheimer’s disease is described by the construction of neuronal plaques and neurofibrillary tangles composed of phosphorylated tau protein. Mitochondrial dysfunction may be a noticeable feature of Alzheimer’s disease and increased production of reactive oxygen species has long been described. Superoxide dismutases (SODs) protect from excess reactive oxygen species to form less reactive hydrogen peroxide. It is suggested that SODs can play a protective role in neurodegeneration. In addition, PI3K/AKT pathway has been shown to play a critical role on the neuroprotection and inhibiting apoptosis via the enhancing expression of the SODs. This pathway appears to be crucial in Alzheimer’s disease because it is related to the tau protein hyper-phosphorylation. Dietary supplementation of several ordinary compounds may provide a novel therapeutic approach to brain disorders by modulating the function of the PI3K/AKT pathway. Understanding these systems may offer a better efficacy of new therapeutic approaches. In this review, we summarize recent progresses on the involvement of the SODs and PI3K/AKT pathway in neuroprotective signaling against Alzheimer’s disease.

## 1. Introduction

Alzheimer’s disease (AD) is the greatest form of dementia, and presently has no effective therapy to improve the symptoms [1,2]. The typical symbol in the diagnosis of AD has been described the increase of neurofibrillary plaques in the brain [3,4]. These aggregates of insoluble protein consist of hyper-phosphorylated tau and abnormal amyloid-β isoforms with some metals such as Fe, Zn, and Cu [5]. Progression of AD can be exacerbated by oxidative stress [6,7]. Brain is an active tissue that depends on oxidative phosphorylation as means for preserving high biological energy [8]. This oxidative phosphorylation occurs in the mitochondria. This process yields by-products of potentially damaging oxygen radicals. Therefore, the ROS are generated during mitochondrial oxidative metabolism as well as in cellular response to pathogens, which act as signaling molecules and regulate numerous physiological processes including differentiation, proliferation, apoptosis, and cell migration [9,10]. Therefore, ROS is suggested as a key determinant of brain health. Eukaryotes have an evolved defense system against such damaging ROS depending on the situation. Chief members of this system are superoxide dismutases (SODs), an enzyme family that efficiently converts superoxide to the less reactive hydrogen peroxide [11]. Loss of SODs activity could result in many pathological phenotypes including neurodegenerative disorder such as AD in the metabolically active tissues [12,13]. 

There are no pharmacologically effective therapies that have been accepted for the management of AD. Options are restricted to symptomatic interventions that slow-down the disease-expansion [14]. Developing new effective therapies for AD requires elucidation of the neuroprotective mechanisms. One of the confirmed mechanisms shown in experimental AD animals involves reducing ROS-induced oxidative damages [15,16]. Intrinsic ROS comes from mitochondria during the procedure of oxidative phosphorylation to yield biochemical energy in the form of ATP. Inflammation induces excessive immune cell accumulation, which has the ability to produce ROS. As inflammation is one of the sources of ROS at the tissue sites it is therefore important for cells to have their inflammation reduced before ROS could damage cellular key macromolecules such as DNA [17]. Clinical findings have shown an upregulation of antioxidant enzymes such as SODs may be effective in the early disease progression of AD [18,19]. The objective of this paper is to review and summarize the studies linking potential protective factors to the pathogenesis of AD, especially focusing on the roles of the cellular signaling pathway affecting the function of SODs. 

## 2. ROS is Involved in the Pathogenesis of AD

**Figure 1:**
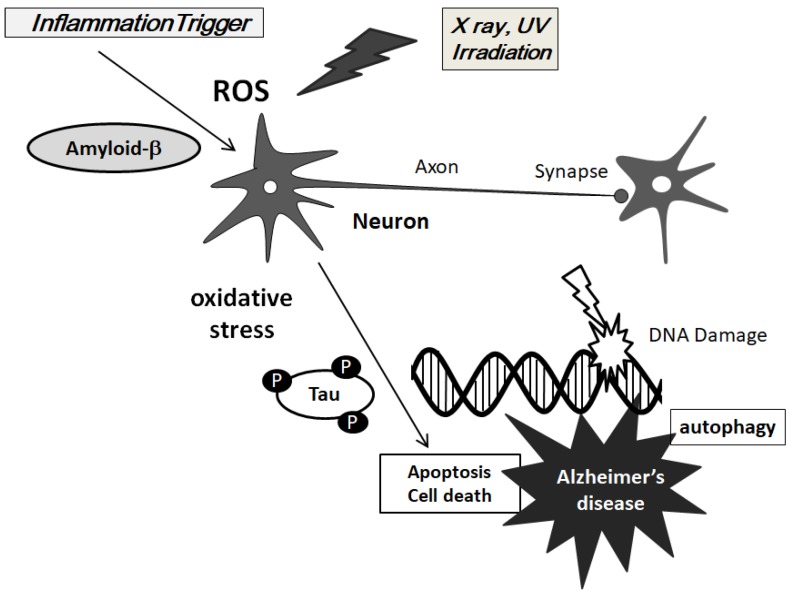
Hypothetical schematic image of the pathogenesis pathway for Alzheimer’s disease is shown. Inflammation trigger, amyloid-β, X-ray, and UV irradiation all contribute to the ROS formation. The uncontrolled generation of ROS and oxidative stress mediated by inflammation and/or amyloid-β might contribute to neuron apoptosis via the hyper-phosphorylation of tau protein. Note that some critical pathways such as NADPH oxidase activation have been omitted for clarity.

The ROS are characterized by a group of oxygen-containing molecules resulting from oxygen metabolism in the cells, which have essential roles in standard physiology and diseases including neurodegenerative diseases and cancer [20]. Under normal physiological situations, they participate in redox reactions, and assist as second messengers for regulatory functions. The key biochemical change in AD is the extreme accumulation of amyloid-β modified by the ROS into toxic products, which progressively aggregate into senile plaques promoting p53 mediated apoptosis [21]. In general, brain function is reduced leading to the enriched plaque formation [22]. Animal models suggest correction plaque formation by decreasing oxidative damage [23]. Oxidative stress also supports a process of progressive failure of autophagy in neurons [24]. It has been reported that abnormal amyloid-β inhibited long-term potentiation in neurons that was saved with administration of an antioxidant [25], suggesting a synergistic achievement between the progression of AD pathogenesis and oxidative stress (Figure 1). Therefore, endogenous oxidative stress may be a precondition for neuronal loss in AD. Other reports show that oxidative stress promotes tau phosphorylation [26]. In addition, ROS causes even tau hyper-phosphorylation via GSK-3β phosphorylation and it might be related to impaired memory deficit [27]. Tau phosphorylation in the proline-rich area inhibits its microtubule-association action and supports its self-aggregation [28,29]. When hyperphosphorylated tau separates from microtubules, increased cytosolic concentrations of phosphorylated tau takes place. Hyperphosphorylated tau is also the main element of the neurofibrillary tangles in degenerating neurons in AD [30]. The tau’s hyperphosphorylation may contribute to neurodegeneration in addition to its aggregates [31].

Reducing oxidative damage in the brain might prevent the loss of neurons over time across multiple neuronal diseases. Surprisingly, it has been suggested that increased ROS production may have an integral role in the development of sporadic AD prior to the appearance of amyloid and tau pathology [32]. Little is known about the primary regulation of signaling molecules by ROS. One mechanism by which ROS are supposed to utilize the effects may be through the reversible regulation of target molecules such as PKC, MAPK, PI3K, and PTEN [33]. However, it is almost unclear how the ROS could stimulate them. Cellular ROS metabolism might be tightly regulated by functional modifications of a variety of key molecules. Most abundant ROS within cells including mitochondria may affect synaptic plasticity and/or memory function [34]. Dysfunction of mitochondria is synergistic to the pathological consequences of AD brain, which have been informed in tissues from patients of AD [35]. Remarkably, interactions with the amyloid-β have been revealed to inhibit the mitochondrial respiration [36]. Anyway, it is obvious that the maintenance of ROS levels in the extracellular and cytosolic situation is essential to maintaining the healthy central nervous system (CNS).

## 3. Characterization of Superoxide Dismutases

**Figure 2:**
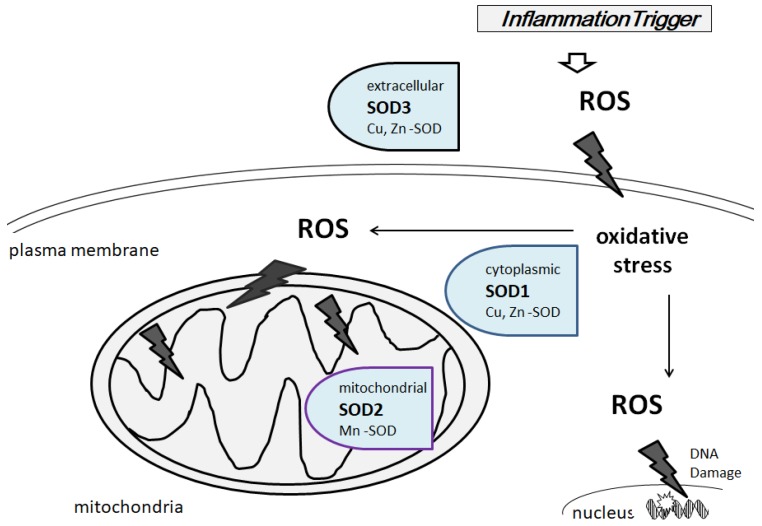
Schematic localization of human SOD1, SOD2, and SOD3 are compartmentalized (SOD1, cytoplasm; SOD2, in the mitochondria; SOD3, in the extracellular space). The lightning bolts show the oxidative attack of ROS on macromolecules such as proteins, lipids, and DNAs.

A group of metal-containing enzymes, named superoxide dismutases (SODs), have a dynamic antioxidant role characterized by their scavenging of ROS [37]. The SODs have been thought the first line of defense system against oxidative stresses, which implies that SOD can play a defensive role in neurodegeneration. Before ROS can oxidize DNA and/or proteins, SODs catalyze the reaction of superoxide to the less reactive hydrogen peroxide. SODs use a metal cation to facilitate their catalysis activity. The presence of metals such as Cu or Zn or Mn or Fe is essential for the function [38]. There are some evidences that changed metal homeostasis in the brain may be the reason for endogenous oxidative stress [39]. Three types of SODs are known in human species (Figure 2). 

The most abundant cytosolic enzyme is Cu/Zn containing SOD1, which can play an imperative role in the avoidance of damage to the CNS [40,41]. Loss of SOD1 upsurges ROS level, which is assumed to trigger oxidative DNA damage to cells. In addition, mutations in the SOD1 gene are responsible for impairment to the mitochondria leading to the beginning of the progressive neurodegenerative diseases such as familial amyotrophic lateral sclerosis [42,43]. It has been shown SOD1-null animals progress some age-related diseases such as muscle atrophy [44]. 

Roles of Mn containing SOD2 enzyme are also serious to neurodegenerative diseases. In addition, SOD2 may be a tumor suppressor, as SOD2 expression has been reported reduced in tumors [45]. SOD2 is located in the mitochondrial matrix, where is the crucial site of free radical creation from the mitochondrial electron transport chain [46]. It seems that SOD2 is required for preserving mitochondrial integrity [47]. A main function of SOD2 might be to protect mitochondrial DNA against oxidative damage. Decreased SOD1 expression has been presented to cause a compensatory escalation in SOD2 [48]. The *SOD2* gene is subjected to regulation by a number of inflammatory cytokines, growth factors, and ultra-violet ray irradiation as well as post-transcriptional epigenetic regulations [49]. One of the significant processes affected by SOD2 is energy metabolism. It has been revealed that SOD2 overexpression makes an increase in ATP creation through vigorous mitochondrial respiration [50]. Mitochondrial ATP production activity has been reported impaired in AD [51] On the contrary, transgenic mice with one deleted copy of the *SOD2* gene show acceleration of AD pathology [50,51]. Interestingly, this reduction in SOD2-function increased amyloidosis in the vasculature of the animal brain [52,53].

The Cu/Zn containing SOD3 is secreted to the extracellular matrix in tissues contained in the CNS [54]. Downregulation of SOD3 has been found to lead DNA copy number change and/or hypermethylation in the promoter region [55]. It has been revealed that overexpressed SOD3 causes an increase of hypoxia inducible factor-1α (HIF1α) in cells [56]. However, induction of vascular endothelial growth factor (VEGF) is suppressed by the SOD3 expression [57], which can contribute in metabolic regulation of cells in the CNS by changing critical blood flow to the brain [58]. Probably, appropriate superoxide scavenging may decrease expression of both VEGF and HIF1α. SOD3, but not SOD2 and SOD1, is induced by several antioxidants such as vitamin C and is regulated through transcription factor NRF2 [59]. Studies indicate that prevention of ROS production by SODs decreases neuronal cell death, blood brain barrier (BBB) disruption, and microglial cell activation, which may have extraordinary therapeutic potential to reduce neuronal cell death [60]. 

## 4. Suggestions of PI3K/AKT/PTEN Signaling on SOD Expression and Pathogenesis of AD

Many studies have shown an antioxidant function for tumor suppressor molecules, activating the expression of several antioxidant genes leading to increased expression of SODs for combating oxidative stress [61,62]. For example, *PTEN* (phosphatase and tensin homolog deleted on chromosome 10) is a tumor suppressor gene that is often deleted and/or mutated in a range of human cancers [63]. Although PTEN has been discovered as a tumor suppressor, PTEN is also involved in several other diseases such as diabetes and AD [64]. Negatively, PTEN controls the activity of PI3K/AKT through exchanging PIP3 to PIP2. The PIP3 is the key second messenger of the PI3K that mediates receptor tyrosine kinase signaling to the AKT cell survival kinase [65]. High levels of PIP3 at plasma membrane stimulate PH domain-containing proteins, such as AKT, resulting in its phosphorylation and activation, which then phosphorylates target proteins involved in cell survival and intracellular metabolism for the cell protection [66]. Consequently, AKT activation may play a therapeutic role in neurodegenerative disorders [67]. Furthermore, PTEN has been documented to play a critical role in neural functions whose level has been shown reduced in AD brains [68]. Presenilins may play an essential role in signaling pathways that are critical for the pathogenesis of AD, regulating induction of the HIF1α [69]. Presenilins are responsible for cleavage of amyloid precursor protein to form amyloid-β. Phosphorylation of presenilin 1 (PS1) leads to activation of the PI3K/AKT survival signaling [70]. Abnormal activation of GSK-3β, a downstream kinase of PI3K/AKT signaling, can reduce neuronal viability [71]. Activated PI3K/AKT phosphorylates various biological substrates including GSK-3β. As GSK-3β has been revealed to phosphorylate tau, dysfunction of PI3K/AKT signaling causes GSK-3β hyper-activity and directs to tau hyper-phosphorylation, an important event in AD pathogenesis [72]. Actually, downregulation of the AKT corresponding to the elevated GSK3β activity may be related to the dysfunctional pathogenesis of AD brain [73]. For example, a study has suggested that PI3K/AKT signaling is attenuated in the brains of AD patients [74]. Moreover, PI3K/AKT inhibitor upsurges tau hyperphosphorylation [75]. 

**Figure 3:**
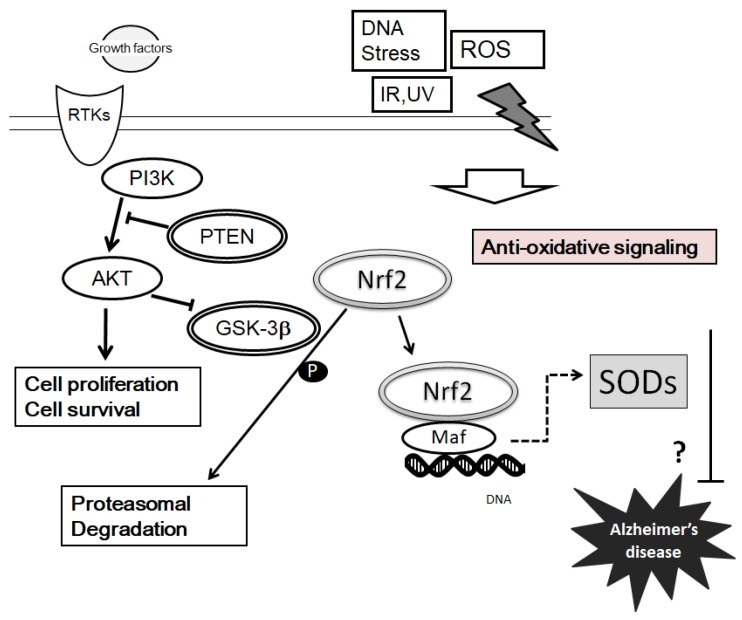
Implication of SODs in the neuroprotection against Alzheimer’s disease via the signaling of PI3K/AKT/PTEN/GSK-3β pathway, and examples of molecules known to act on the regulatory pathways are shown. PI3K/AKT/PTEN/GSK-3β pathway and SODs may contribute to neuroprotection in AD. Hammerheads mean inhibition. Note that some critical events have been omitted for clarity

PI3K/AKT/PTEN pathway acts as a fundamental factor of cell fate regarding cell apoptosis and senescence, which is mediated by intracellular ROS production [76]. The pathway signaling protects cells against oxidative damage via the Nrf2 activation that is a transcription factor [77]. After translocation into nucleus, the Nrf2 creates a heterodimer with other transcription factors such as Maf protein (Figure 3), which in turn binds to the DNA regulatory sequence termed antioxidant response elements (ARE). ARE is located in the promoter region of several genes encoding various antioxidant enzymes [78]. Silencing of Nrf2 significantly inhibits mRNA and protein expression of SOD1, and SOD2 [79]. We suppose that degradation of Nrf2 via the phosphorylation by GSK-3β may lead to a similar result (Figure 3). In addition, the Nrf2-Maf complex level is reduced by oxidative stress [80]. On the contrary, AKT-mediated Nrf2 activation by resveratrol-treatment attenuates oxidative stress and cellar apoptosis [81], which may contribute to the neuroprotection. Upon long oxidative stress exposure, the enzymatic activity of an ubiquitin E3 ligase complex is inhibited and the complex fails to degrade Nrf2, resulting in the transcriptional activation of Nrf2 target genes [82]. Presumably, it seems to be a cell protective feedback from the oxidative stress.

## 5. Diet Approach for Neuronal Cellular Protection in AD Prevention

Due to an absence of actual treatments, the brain dysfunction in AD is a public health anxiety. Therefore, a number of preventive factors have been proposed by epidemiological research including modifiable lifestyle factors such as diet. It has been revealed that dietary choices can play a certain role in the neuroprotection of AD [83]. However, the relation between nutrient consumption and neuroprotection is fairly complex. In addition, the convolution of the human diet makes it difficult to examine their distinct effects. Although many lifestyle factors affect brain function, some involvements of foods might be promising in the prevention of brain dysfunction. The many properties of foods could have some cell protective potential, which could be facilitated through efficient modulation of the PI3K/AKT/PTEN signaling pathway. For example, insulin receptor signaling may circuitously contribute to long-term memory integration and improved spatial learning [84], and insulin treatment improves memory and cognitive function in patients with AD [85]. The neuroprotective effects of insulin are mediated in part by the PI3K/AKT/PTEN pathway [86]. Curcumin, a component of turmeric, potently depresses the levels of amyloid-β in a dose-dependent manner. Furthermore, studies have indicated that curcumin can improve the pathology related to the amyloid-β in experimental animal models [87]. Curcumin can recover structure and plasticity of synapses, and can enhance the host’s learning and memory abilities [87,88]. It is probable that the neuroprotection of curcumin is mediated via the PI3K/AKT signaling. Curcumin effectively inhibits oxidative stress via a mechanism involving the Nrf2 and PI3K/AKT signaling pathway [89]. Dietary treatment with curcumin also has the potential to recover phospho-tau pathology [90]. 

Intestinal fat absorption increases the synthesis and secretion of apoliprotein A-IV (apo A-IV), which considerably activates PI3K pathway [91]. In addition, the n-3 polyunsaturated fatty acids (PUFAs) are a family of biologically active fatty acids, which have a variety of biological roles that relate to cellular functions. A member of this family, α-linolenic acid, can be transformed to the long-chain n-3 PUFAs such as eicosapentaenoic acid (EPA) and docosahexaenoic acid (DHA). Using of n-3 PUFAs has been shown as a possible preventive portion for AD [92]. Neuroprotection by inhibiting PTEN tumor suppressor with food ingredients has been described by activating the anti-apoptotic PI3K/AKT signaling pathway in neuronal cells [93]. For example, rosemary extract represses *PTEN* expression in culture cells [94]. Furthermore, activated PPARs regulate expression of the *PTEN* [95]. As PTEN is induced by the activated PPARs, this may also suggest a potential therapeutic character for the management of those PI3K/AKT/PTEN-related diseases. A wide variety of compounds have been identified as PPARs ligand including the n-3 PUFAs [96], which have a valuable effect on most of the metabolic risk factors [97]. Linoleic acid could also bind PPARδ quite well [98]. As mentioned before, resveratrol appears to be beneficial as an anti-AD agent. In addition, resveratrol treatment prevents the pro-inflammatory effect of amyloid-β on macrophages [99]. 

Other than that, genistein, a phytoestrogen present in soy, has a strong estrogen-like effect, which is favorable for plasticity of AD [100]. Active genistein aglycone treatment may inhibit ROS production enhancing the activities of SODs [101]. Lycopene inhibits apoptosis by reducing ROS, and by inhibiting mitochondrial dysfunction [102]. It has been suggested that dietary intake of copper stabilizes SOD1 activity and attenuates amyloid-β production in the AD mouse model, indicating therapeutic benefit [103]. Taken together, neuro-protection in AD could be performed by certain diets.

## 6. Future Perspectives

Diet generally consists of complex combinations of lipids and/or nutrients that might work synergistically or antagonistically. Curcumin and n-3 PUFAs are considered as potentially exerting effects on some different cellular levels. Molecules involved may be regulated at another multiple levels including gene transcription, protein stability, phosphorylation, and so forth. As shown here, several molecules have been implicated in forms of AD, principally relating to amyloid processing. Looking forward, a detailed understanding these regulations is critical for diet therapeutic intervention and the effective design of novel therapeutics. Especially, it will be important to understand the mechanism-of-action, which will lay a foundation for targeting SODs in appropriate AD stages. It is likely that AD therapy has to be personalized and stage specific. As ROS are implicated in a wide-ranging of pathological processes, then it seems reasonable to speculate that constitutive over expression of SODs genes must be protective against AD. However, the contribution of SODs to the pathogenesis of diseases has long been controversial. For example, it was found that in the context of the AD animal model, the overexpression of SOD2 is protective, and can prevent some of the pathological features characteristic of that animal [104,105]. On the contrary, there are some reports where SOD2 could not improve and/or protect against neuronal decline in experimental animals. Further molecular studies are required to recognize the exact mechanisms and to determine the roles regarding the conservation of brain health. In addition, long-term studies are mandatory to clarify the effect of treatment in the management of AD. It is also mandatory to explore the combination with other therapies that are known to reduce ROS levels, and could improve consequences. 

## Abbreviations

AD	Alzheimer’s disease
Apo A-IV	apoliprotein A-IV
ARE	antioxidant response elements
ATF2	activating transcription factor 2
ATP	adenosine triphosphate
BBB	blood brain barrier
CNS	central nervous system
DHA	docosahexaenoic acid
DNA	deoxyribonucleic acid
EPA	eicosapentaenoic acid
GSK3β	glycogen synthase kinase-3β
HIF1α	hypoxia inducible factor-1α
Maf	macrophage activating factor
MAPK	mitogen-activated protein kinase
Nrf2	NF-E2-related factor-2
PIP3	phosphatidylinositol 3,4,5-triphosphate
PIP2	phosphatidylinositol 4,5- bisphosphate
PI3K	phosphoinositide-3 kinase
PKC	protein kinase c
PPAR	peroxisome proliferator-activated receptor
PPREs	PPAR response elements
PS	presenilin
PTEN	phosphatase and tensin homologue deleted on chromosome 10
PUFAs	polyunsaturated fatty acids
ROS	reactive oxygen species
SODs	superoxide dismutases
